# Supplementary material for: Reactivation of a somatic errantivirus and germline invasion in Drosophila ovaries
Source: Nat Commun. 2023 Sep 29;14:6096. doi: 10.1038/s41467-023-41733-5 (PMC10541861; doi:10.1038/s41467-023-41733-5)
Supplement: Supplementary file 1 — Supplementary Information [file 41467_2023_41733_MOESM1_ESM.pdf]

Supplementary Information

Reactivation of an errantivirus in *Drosophila* ovarian somatic tissue: from germline invasion to taming

Marianne Yoth[1], Stéphanie Maupetit-Méhouas[1], Abdou Akkouche[1], Nathalie Gueguen[1], Benjamin Bertin[2], Silke Jensen[1]*, Emilie Brasset[1]*

[1] iGReD, Université Clermont Auvergne, CNRS, INSERM, Faculté de Médecine, 63000 Clermont-Ferrand, France.

[2] LIMAGRAIN EUROPE, Centre de recherche, 63720 Chappes, France.

* Correspondence: emilie.brasset@uca.fr, silke.jensen@uca.fr

Supplementary Figures

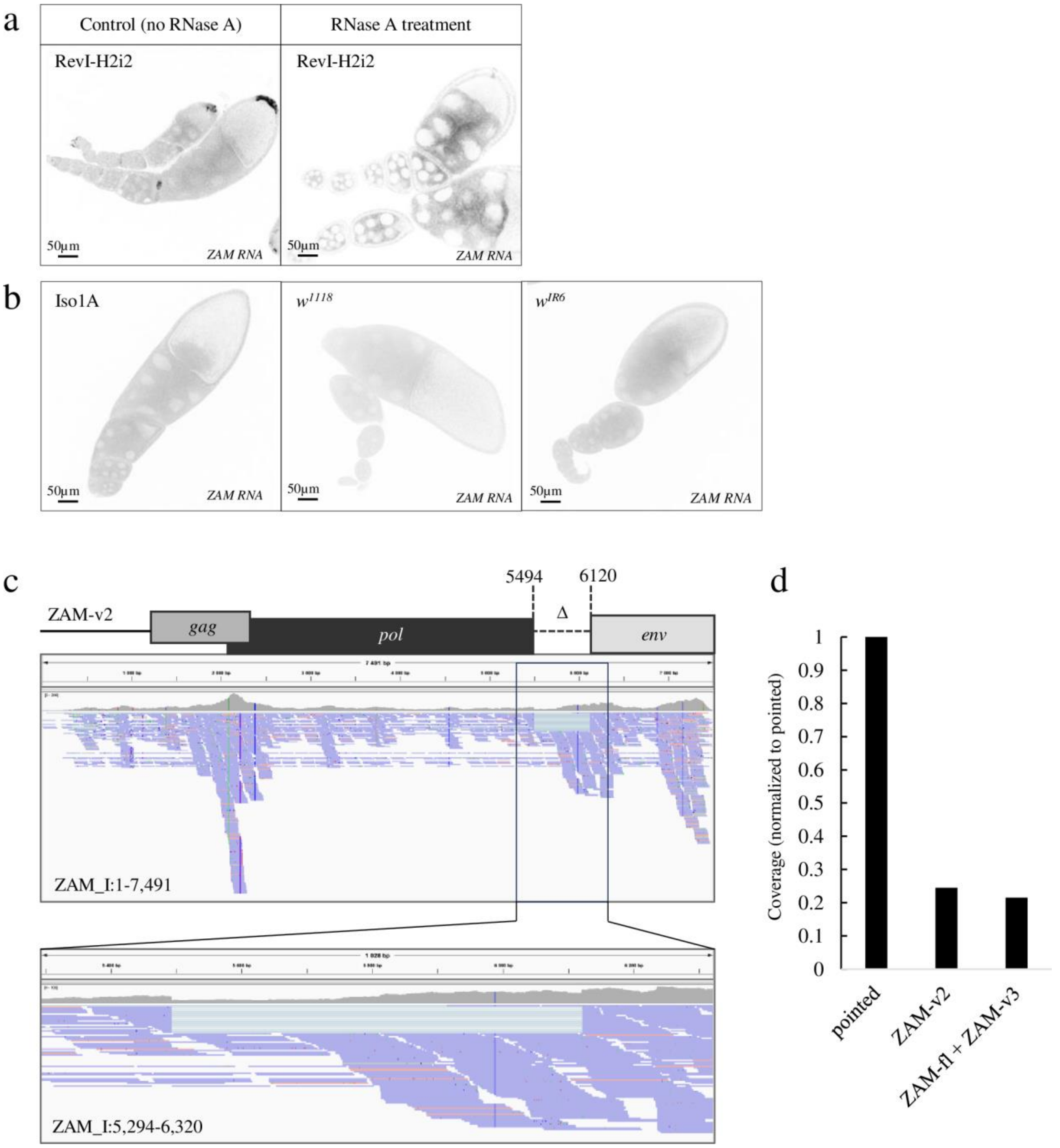

Supplementary Figure 1

This Figure is related to Figure 1. **a.** Color-inverted confocal images of ovarioles from RevI-H2i2 ovaries showing *ZAM* RNA signal without (left panel) and after (right panel) RNase A treatment. **b.** Color-inverted confocal images of ovarioles showing that *ZAM* RNA is completely absent in Iso1A, w^{1118}, and w^{IR6} ovaries. **c.** Mapping of RNA-Seq reads of the full-length *ZAM* (ZAM-fl) and of ZAM-v2 to the reference *ZAM* internal sequences *ZAM*_I (Repbase) allowing splitting of the reads. Above is shown ZAM-v2 structure. The inset below shows a zoom on the ZAM-v2 deletion with the corresponding split reads. Reads corresponding to *ZAM* RNA are in blue, antisense reads are in red. Positions of the shown regions within ZAM_I are indicated. d. Coverage of RNA-seq reads corresponding to the indicated *ZAM*-variants normalized to the average coverage of the gene *pointed*.

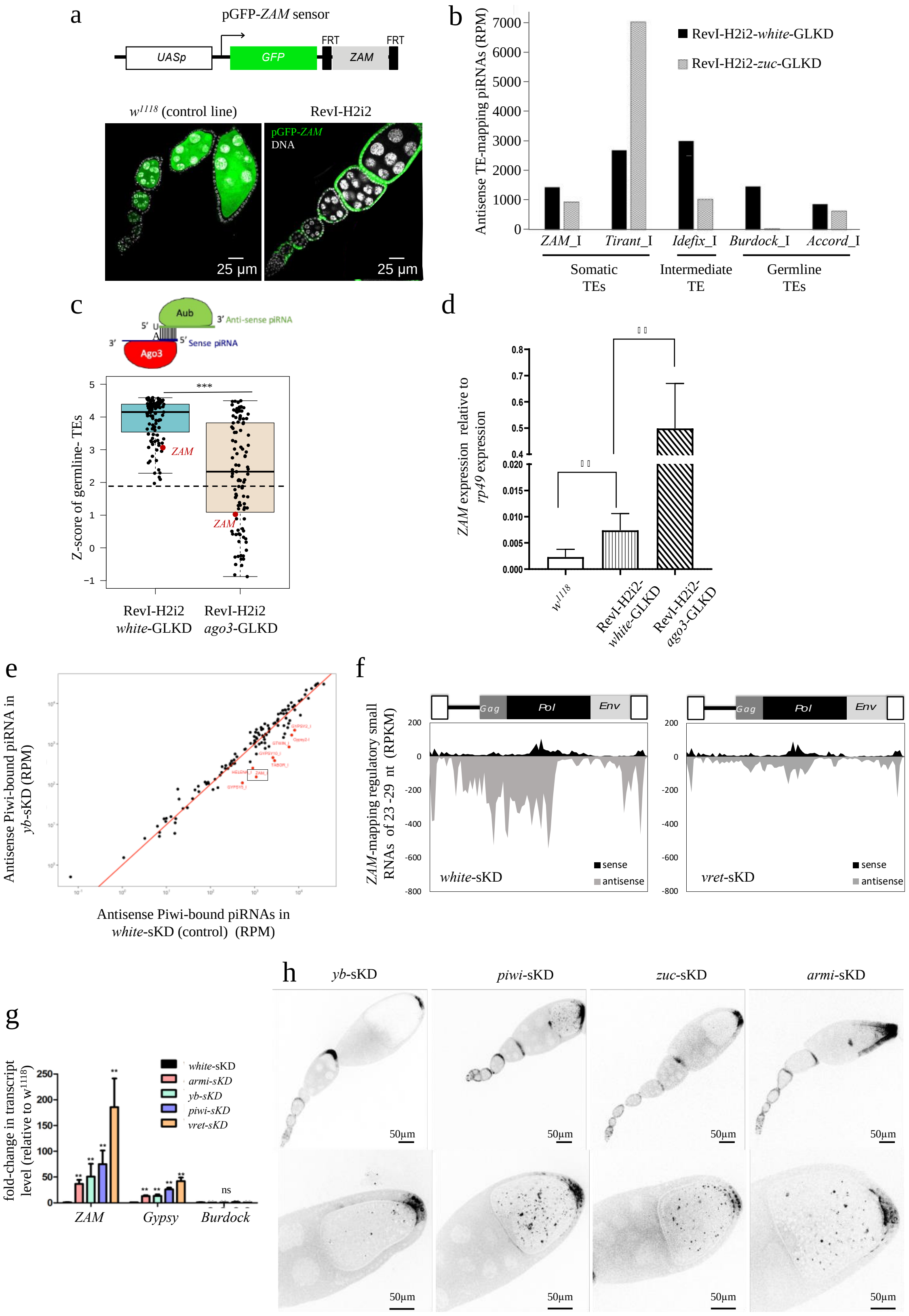

Supplementary Figure 2

This Figure is related to Figure 2. **a**. Confocal images of ovarioles with GFP (green) and DNA (white) staining. Ovarioles of the progeny of a cross between either *w*^{1118} control females or RevI-H2i2 females and males carrying the pGFP-*ZAM* sensor transgene driven by actin-Gal4. In *w*^{1118} control ovaries, the transgene was completely silenced in somatic cells and strongly

expressed in germline cells Conversely, in RevI-H2i2 ovaries, the transgene was silenced in the germline and strongly expressed in somatic cells indicating that *ZAM*-derived piRNAs produce in the RevI-H2i2 germline cells efficiently guide sensor silencing in these cells. **b.** Antisense regulatory piRNAs (23-29 small-RNAs complexed with Argonaute proteins) mapping to TE internal sequences (0-3 mismatches) in the RevI-H2i2 line upon *white-* (control), or *zuc*-GLKD. Normalized per million of genome-mapping piRNAs. **c.** Box plot displaying the Z-score distribution of 10nt 5'-overlaps for TE-mapping piRNAs in the *white*-GLKD and *ago3*-GLKD RevI-H2i2 lines. Each dot represents a TE with a Z-score >1.96 (dotted line) in the control condition (*white*-GLKD). *ZAM* is highlighted in red. The midline indicates the median value, and the box shows the first and third quartile. Error bars indicate the SD. ****p-value* <0.001 (Mann-Whitney test). **d**. Bar plot showing the steady-state *ZAM* RNA levels in total RNA from ovaries with the indicated genotypes (RT-qPCR, normalized to *rp49*, primer sequences in Supplementary Table S5). At least three biological replicates and two technical replicates were used. ***p-value* <0.01 (Mann-Whitney tst); error bars indicate the SD. **e**. Scatter plot showing the normalized counts of antisense Piwi-bound piRNAs mapping to individual internal TE sequence in control ovaries (*white*-sKD) versus *yb*-sKD ovaries. Antisense piRNA counts, mapped allowing up to 3 mismatches, were normalized per million of genome-mapping piRNAs (RPM, here in logarithmic scale). TEs in blue have a *yb*-sKD/*white*-sKD ratio >3; TEs in red have a ratio <0.3. *ZAM* is boxed in black. **f.** Density plot along the *ZAM* sequence of *ZAM*-mapping regulatory piRNAs produced in *white-* and *vret*-sKD ovaries (up to 3 mismatches). **g**. Fold-change in the steady-state *ZAM, Gypsy* and *Burdock* RNA level for the indicated sKD ovaries, compared with *white*-sKD ovaries, measured by RT-qPCR (primer sequences in Supplementary Table S5). At least three biological replicates and two technical replicates were used. ***p-value* <0.01 (Mann-Whitney test); error bars indicate the SD. **h**. Color-inverted confocal images of ovarioles (upper panels) and stage 10 egg chambers (lower panels) from the indicated sKD lines showing *ZAM* RNA expression in follicle cells and in the ooplasm in indicated conditions.

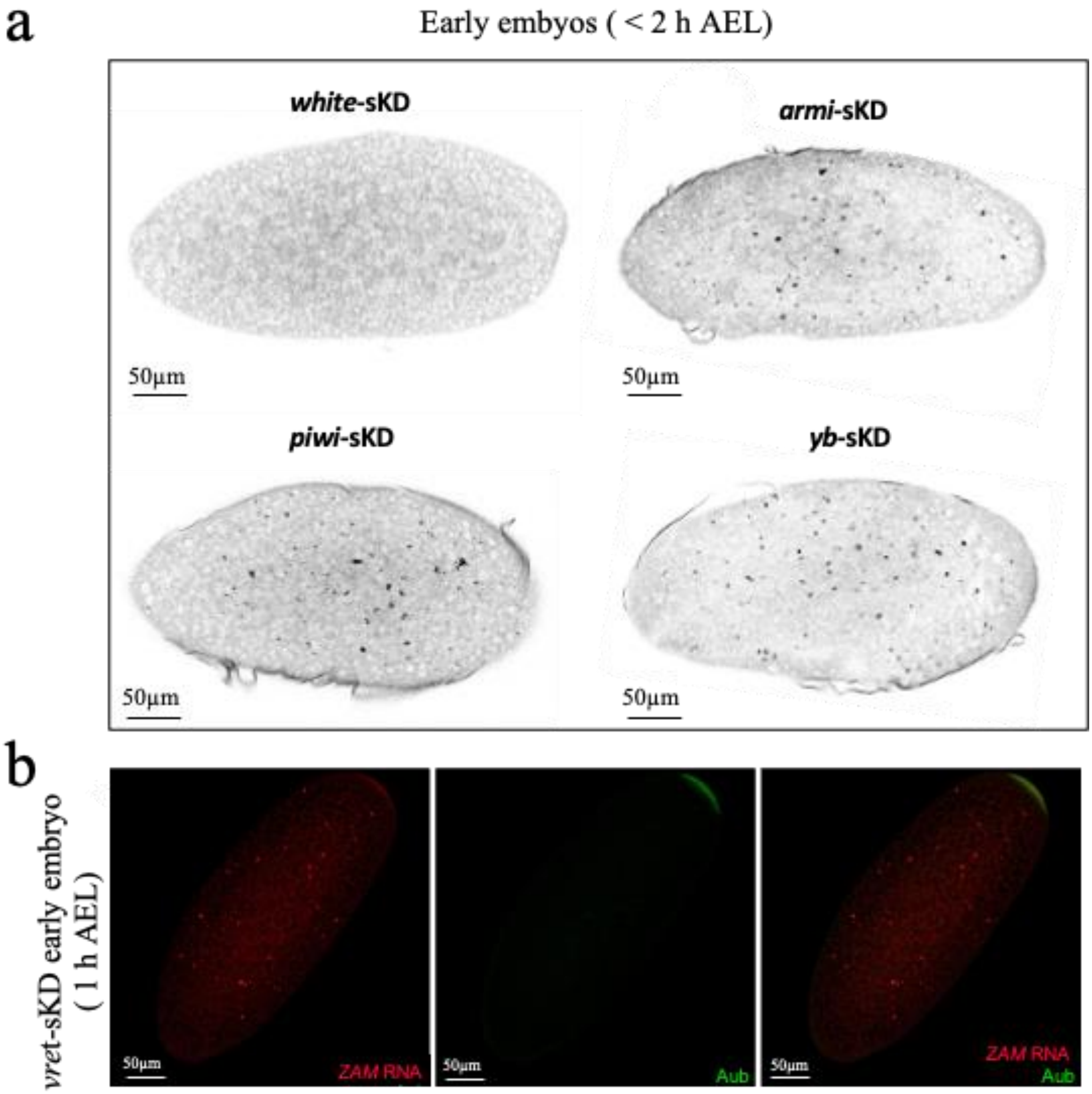

Supplementary Figure 3

This Figure is related to Figure 3. **a.** Color-inverted confocal images of early embryos collected 0-2 hours after egg laying (AEL). Mothers harbor the indicated sKD of the piRNA pathway. *ZAM* RNA signal was detected by smRNA FISH. **b.** Projection of confocal images of stage 1 embryos (0-1 h AEL) laid by *vret*-sKD females showing *ZAM* RNA detected by smRNA FISH and Aub protein immunostaining. Both accumulated at the posterior pole of the embryo where future germ cells cellularize (Z-projection of 25 stacks).

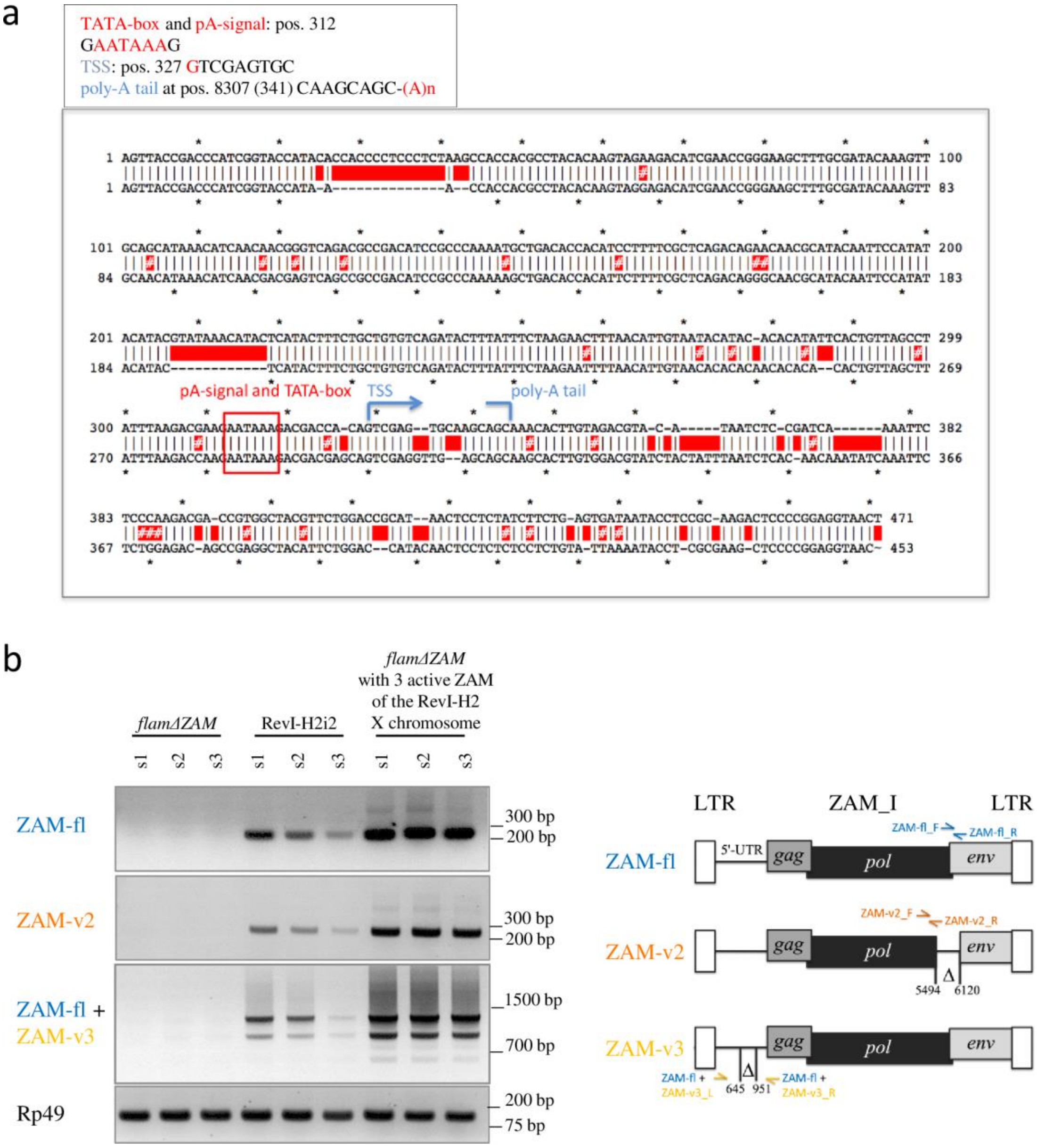

Supplementary Figure 4

This Figure is related to Figure 4. **a.** Sequence alignment of the RevI-H2 consensus *ZAM* LTR and the *ZAM* solo-LTR that remains in the *flam*Δ*ZAM* line after CRISPR-Cas9 deletion of the internal *ZAM* sequence in *flamenco*. The image shows the RevI-H2 consensus *ZAM* LTR (upper sequence) and the *ZAM* solo-LTR (lower sequence) with their TATA-box and polyadenylation signal (pA-signal) at position 312 of the *ZAM* consensus, the transcription start site (TSS, at position 327), the start of the poly-A tail at position 341 (CAAGCAGC-(A)n), according to Leblanc et al, 1997. **b.** RT-PCR results showing the expression of the different *ZAM*-variants in the ovaries of the following fly lines: *flam*Δ*ZAM*, RevI-H2i2 and *flam*ΔZAM with three copies of *ZAM* from the RevI-H2 X chromosome (one ZAM-fl, one ZAM-v2 and one ZAM-v3). Three biological replicates were used for each condition (s1, s2, s3). *Rp49* has been used as the housekeeping gene control. The primers used for specific amplification of each variant are indicated on the schematic representation of *ZAM* variants and represented by arrows, and color-coded as follows: Blue: Primers for specific amplification of ZAM-fl. Orange: Primers for specific amplification of ZAM-v2 (primer ZAM-v2_R overlaps left and right edges of the ZAM-v2 deletion). Yellow: Primers for amplification of ZAM-fl and ZAM-v3, where the size of the amplification product differs due to a specific deletion in the 5'-UTR of ZAM-v3. (primer sequences in Supplementary Table S5).

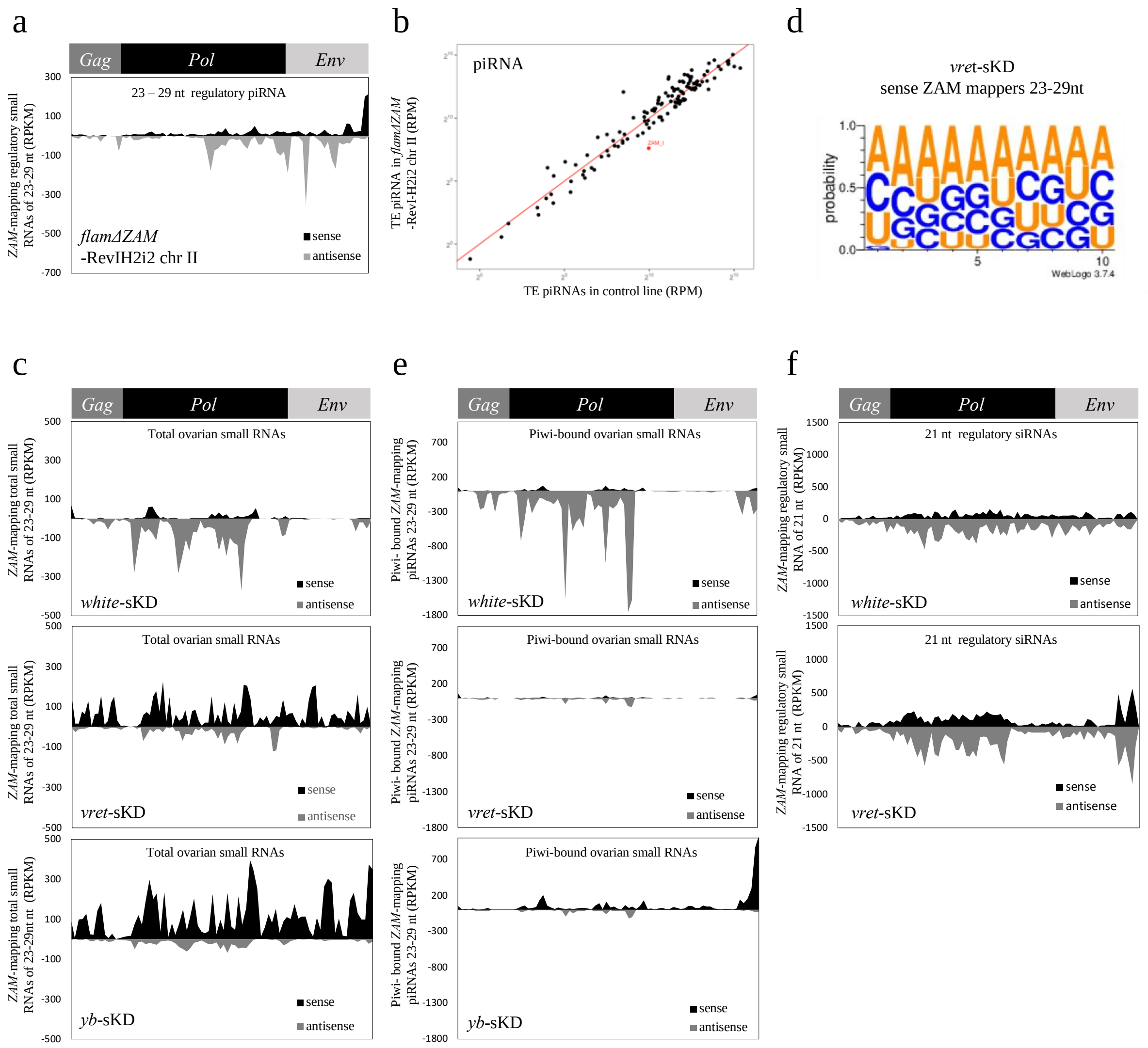

Supplementary Figure 5

This Figure is related to Figure 5. **a**. Density plot of *ZAM*-mapping regulatory piRNAs (23-29 nt small-RNAs complexed with Argonaute proteins) along the *ZAM* internal sequence in *flamΔZAM*-RevI-H2i2 chr II ovaries (up to 3 mismatches). **b.** Scatter plots showing the normalized counts of regulatory piRNAs mapping to individual internal TE sequences in control ovaries (*white*-sKD) versus *flamΔZAM*-RevI-H2i2 chr II ovaries. Antisense piRNA counts, mapped allowing up to 3 mismatches, were normalized per million of genome-mapping piRNAs (RPM, here in logarithmic scale). **(c)** Density plot of total small RNAs of 23 to 29 nt along the *ZAM* sequence in *white*-sKD, *vret*-sKD and *yb*-sKD ovaries (up to 3 mismatches). **c.** Density plot of total *ZAM*-mapping small RNAs of 23 to 29 nt along the *ZAM* internal sequence in *white*-sKD, *vret*-sKD and *yb*-sKD ovaries (up to 3 mismatches). **d**. Sequence logo for the first ten positions in *ZAM*-mapping sense small RNAs of 23 to 29nt (from total ovarian small RNA) in *vret*-sKD ovaries. The nucleotide height represents its relative frequency at that position. **e.** Density plot of *ZAM*-mapping Piwi-bound piRNAs along the *ZAM* internal sequence in *white*-sKD, *vret*-sKD and *yb*-sKD ovaries (up to 3 mismatches). **f.** Density plot of *ZAM*-mapping regulatory siRNAs (21nt small-RNAs complexed with Argonaute proteins) along the *ZAM* internal sequence produced in *white*-sKD and *vret*-sKD ovaries (0-1 mismatch).

Cross to establish a line RevIH2-i2 with the Nanos-gal4 driver

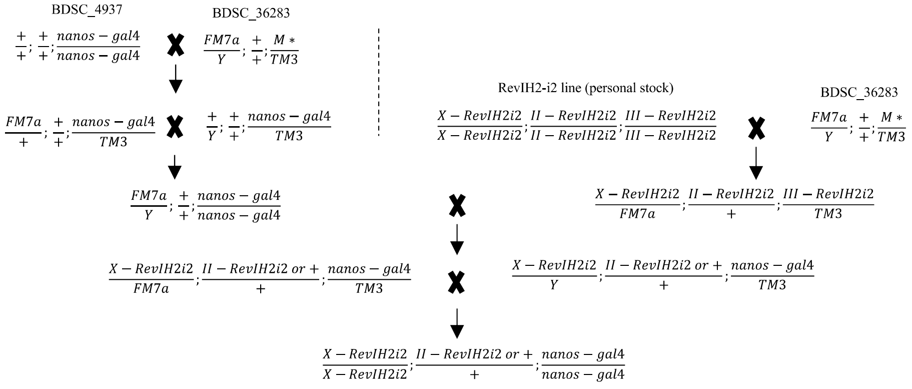

Cross to establish a line RevIH2-i2 with nanos-gal4 and a shRNA of RNAi

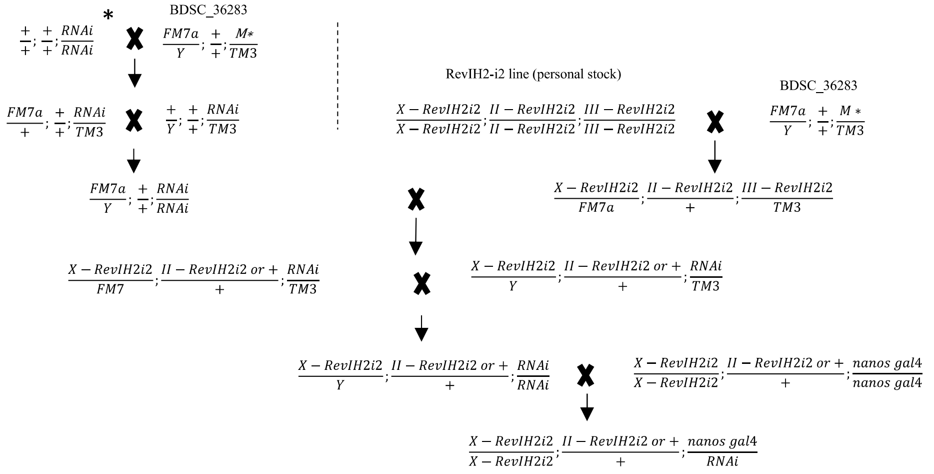

* shRNA of RNAi located on the chromosome III = *white*-sh (BDSC_35573) , *vret*-sh (VDRC_34897) , *armi*-sh (BDSC_34789) , *zuc*-sh (BDSC_35227), *Ago3*-sh (BDSC_35232)

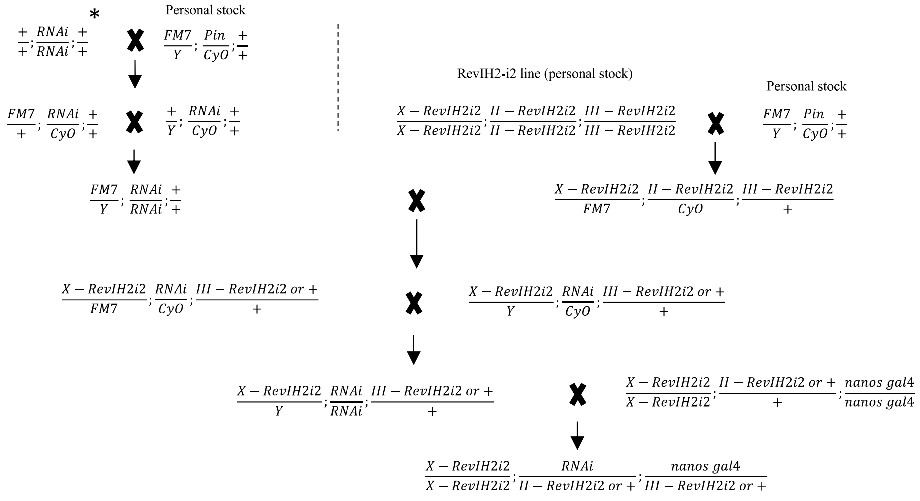

* shRNA of RNAi located on the chromosome II = *piwi*-sh (BDSC_37483)

M* = PBac{w[+mC]=PB}CG11583[c01124] P{ry[+t7.2]=neoFRT}80B
FM7a = Bar^{1} sc^{8} v^{Of} w^{a} y^{31d}
Pin = $\beta Tub60D^{Pin-1}$

Supplementary Figure 6

Description of the genetic crosses performed to obtain the different RevI-H2i2-GLKD lines.
