# Supplementary material for: Reactivation of a somatic errantivirus and germline invasion in Drosophila ovaries
Source: Nat Commun. 2023 Sep 29;14:6096. doi: 10.1038/s41467-023-41733-5 (PMC10541861; doi:10.1038/s41467-023-41733-5)
Supplement: Supplementary file 3 — Description of Additional Supplementary Files [file 41467_2023_41733_MOESM3_ESM.pdf]

# Description of Additional Supplementary Files

**Supplementary Data 1:** List of ZAM insertions identified in the RevI-H2i2 line

**Supplementary Data 2:** List of Drosophila lines used in the study

**Supplementary Data 3:** List of smRNA FISH probes used in the study

**Supplementary Data 4:** List of antibodies used in the study

**Supplementary Data 5:** List of primers used in the study

**Supplementary Data 6:** List of all small RNA sequencing performed in the study

**Supplementary Data 7:** List of chemicals and reagents used in the study

**Supplementary Movie 1**: Confocal Z-stacks overlay of stage 10 egg chambers from *vret* sKD ovaries expressing Yolk-protein-1-GFP (green) showing *ZAM* smRNA FISH signal (red).
