# Supplementary material for: Reactivation of a somatic errantivirus and germline invasion in Drosophila ovaries
Source: Nat Commun. 2023 Sep 29;14:6096. doi: 10.1038/s41467-023-41733-5 (PMC10541861; doi:10.1038/s41467-023-41733-5)
Supplement: Supplementary file 6 — Reporting Summary [file 41467_2023_41733_MOESM6_ESM.pdf]

nature portfolio

Corresponding author(s): Emilie Brasset
Silke Jensen

Last updated by author(s): Sep 6, 2023

Reporting Summary

Nature Portfolio wishes to improve the reproducibility of the work that we publish. This form provides structure for consistency and transparency in reporting. For further information on Nature Portfolio policies, see our Editorial Policies and the Editorial Policy Checklist.

Statistics

For all statistical analyses, confirm that the following items are present in the figure legend, table legend, main text, or Methods section.

n/a	Confirmed	
☐	☒	The exact sample size (*n*) for each experimental group/condition, given as a discrete number and unit of measurement
☐	☒	A statement on whether measurements were taken from distinct samples or whether the same sample was measured repeatedly
☐	☒	The statistical test(s) used AND whether they are one- or two-sided<br>*Only common tests should be described solely by name; describe more complex techniques in the Methods section.*
☒	☐	A description of all covariates tested
☒	☐	A description of any assumptions or corrections, such as tests of normality and adjustment for multiple comparisons
☐	☒	A full description of the statistical parameters including central tendency (e.g. means) or other basic estimates (e.g. regression coefficient) AND variation (e.g. standard deviation) or associated estimates of uncertainty (e.g. confidence intervals)
☐	☒	For null hypothesis testing, the test statistic (e.g. *F*, *t*, *r*) with confidence intervals, effect sizes, degrees of freedom and *P* value noted<br>*Give P values as exact values whenever suitable.*
☒	☐	For Bayesian analysis, information on the choice of priors and Markov chain Monte Carlo settings
☒	☐	For hierarchical and complex designs, identification of the appropriate level for tests and full reporting of outcomes
☒	☐	Estimates of effect sizes (e.g. Cohen's *d*, Pearson's *r*), indicating how they were calculated

Our web collection on statistics for biologists contains articles on many of the points above.

Software and code

Policy information about availability of computer code

Data collection	*Provide a description of all commercial, open source and custom code used to collect the data in this study, specifying the version used OR state that no software was used.*
Data analysis	In-depth study of small RNA using sRNAPipe (Galaxy Version 1.2) (Pogorelcnik et al, 2018) for mapping to the various genomic sequence categories of the D. melanogaster genome (Release 6).<br>Scatter plots were done with RStudio (Version 2023.06.0+421)<br>RNAseq data were analyzed on the local Galaxy platform using HISAT2 alignment (Galaxy Version 2.1.0)

For manuscripts utilizing custom algorithms or software that are central to the research but not yet described in published literature, software must be made available to editors and reviewers. We strongly encourage code deposition in a community repository (e.g. GitHub). See the Nature Portfolio guidelines for submitting code & software for further information.

Data

Policy information about availability of data

All manuscripts must include a data availability statement. This statement should provide the following information, where applicable:

- Accession codes, unique identifiers, or web links for publicly available datasets
- A description of any restrictions on data availability
- For clinical datasets or third party data, please ensure that the statement adheres to our policy

We provided a complete data availability statement in the manuscript: "All sequencing data generated in this study have been deposited in NCBI GEO database, Sequence Read Archive, under accession code GSE213456 for the ONT genome sequencing data, GSE213368 for the small-RNA sequencing data and GSE235966 for the RNA sequencing data. Source data are provided as a Source Data file."

The accession numbers are publicly available.

We provided a complete data availability statement in the manuscript: "All sequencing data generated in this study have been deposited in NCBI GEO database, Sequence Read Archive, under accession code GSE213456 for the ONT genome sequencing data, GSE213368 for the small-RNA sequencing data and GSE235966 for the RNA sequencing data. Source data are provided as a Source Data file."

The accession numbers are publicly available.

We provided a complete data availability statement in the manuscript: "All sequencing data generated in this study have been deposited in NCBI GEO database, Sequence Read Archive, under accession code GSE213456 for the ONT genome sequencing data, GSE213368 for the small-RNA sequencing data and GSE235966 for the RNA sequencing data. Source data are provided as a Source Data file."

The accession numbers are publicly available

Research involving human participants, their data, or biological material

Policy information about studies with human participants or human data. See also policy information about sex, gender (identity/presentation), and sexual orientation and race, ethnicity and racism.

Reporting on sex and gender	N/A
Reporting on race, ethnicity, or other socially relevant groupings	N/A
Population characteristics	N/A
Recruitment	N/A
Ethics oversight	N/A

Note that full information on the approval of the study protocol must also be provided in the manuscript.

Field-specific reporting

Please select the one below that is the best fit for your research. If you are not sure, read the appropriate sections before making your selection.

☒ Life sciences ☐ Behavioural & social sciences ☐ Ecological, evolutionary & environmental sciences

For a reference copy of the document with all sections, see nature.com/documents/nr-reporting-summary-flat.pdf

Life sciences study design

All studies must disclose on these points even when the disclosure is negative.

Sample size	As the number of flies to be used in the experiments was not a limiting factor, no statistical power analyses were used to predetermine sample sizes. Sample sizes were chosen according to conventions in our field for standard sample sizes, as well as published precedents for similar experiments. Thus, for each RNA and DNA extraction, a pool of 50 to 100 flies was used to avoid inter-individual variations and to have a representation at the population scale. The same number of flies was used for sterility tests. For smRNA FISH staining, at least 10 pairs of ovaries were dissected for each experiment and repeated at least 3 times, with similar results obtained each time
Data exclusions	No data exclusion
Replication	When possible, several complementary methods were used to independently test a given hypothesis in parallel (e.g. RT-qPCR , RNAseq, smRNA FISH). All experiments were repeated with biological replicates and, for RT-qPCR, we also included technical replicates that were performed over several days to confirm reproducibility. All replication attempts were successful, except when caused by technical issues (NGS librairies)
Randomization	For the different biological replicates, flies of the same genotype were randomly separated into groups of the same size

Blinding	The investigator was not blind to the flies' genotype during data acquisition or analyses. Genotype blinding was unnecessary for all the sequencing experiment. The same published pipeline "sRNAPipe" (Pogorelcnik et al, 2018) was equally applied regardless of the flies' genotype. For microscopy imaging, initial parameter settings were established using a control line, and these same acquisition parameters were applied to all conditions. Blinding doesn't need to be performed because it can't interfere with the analysis

Behavioural & social sciences study design

All studies must disclose on these points even when the disclosure is negative.

Study description	N/A
Research sample	N/A
Sampling strategy	N/A
Data collection	N/A
Timing	N/A
Data exclusions	N/A
Non-participation	N/A
Randomization	N/A

Ecological, evolutionary & environmental sciences study design

All studies must disclose on these points even when the disclosure is negative.

Study description	N/A
Research sample	N/A
Sampling strategy	N/A
Data collection	N/A
Timing and spatial scale	N/A
Data exclusions	N/A
Reproducibility	N/A
Randomization	N/A
Blinding	N/A

Did the study involve field work? ☐ Yes ☒ No

Field work, collection and transport

Field conditions	N/A
Location	N/A
Access & import/export	N/A
Disturbance	N/A

Reporting for specific materials, systems and methods

We require information from authors about some types of materials, experimental systems and methods used in many studies. Here, indicate whether each material, system or method listed is relevant to your study. If you are not sure if a list item applies to your research, read the appropriate section before selecting a response.

Materials & experimental systems

n/a	Involved in the study
[ ]	[x] Antibodies
[x]	[ ] Eukaryotic cell lines
[x]	[ ] Palaeontology and archaeology
[ ]	[x] Animals and other organisms
[x]	[ ] Clinical data
[x]	[ ] Dual use research of concern
[x]	[ ] Plants

Methods

n/a	Involved in the study
[x]	[ ] ChIP-seq
[x]	[ ] Flow cytometry
[x]	[ ] MRI-based neuroimaging

Antibodies

Antibodies used

Primary antibody
Rabbit anti-Aubergine (Homemade (Eurogentec) - polyclonal antibody
Goat anti-GFP abcam ab450
Rabbit anti-Gag-ZAM Homemade
Rabbit anti-Env-ZAM Homemade

Secondary antibody
Alexa Fluor 488 donkey anti-goat IgG (H+L) invitrogen (Fisher) A11055
Donkey anti-rabbit Alexa 488 IgG (H+L) 2mg/ml life tech - A21206
Goat anti-rat Cy3 Jackson Immunoresearch - 112-165- 143

Validation

The anti-Aubergine antibody has been generated for this paper but targets the same epitope as an already validated antibody (Brennecke, J., Aravin, A.A., Stark, A., Dus, M., Kellis, M., Sachidanandam, R., Hannon, G.J., 2007. Discrete Small RNA-Generating Loci as Master Regulators of Transposon Activity in Drosophila. Cell 128, 1089–1103). We also observed the same expression profile as that observed in different publications using anti-Aubergine antibodies.
Anti-Gag-ZAM and anti-Env-ZAM antibodies have been generated in the laboratory and validated in a previously published study (Leblanc, P., Desset, S., Giorgi, F., Taddei, A.R., Fausto, A.M., Mazzini, M., Dastugue, B., Vaury, C., 2000. Life Cycle of an Endogenous Retrovirus, ZAM, in Drosophila melanogaster. J. Virol. 74, 10658–10669). We observed a similar expression profile for the Gag and Env proteins using the same RevIH2 and wIR6 Drosophila lines.
The anti-GFP antibody has been ordered from Abcam and validated using positive controls, such as cells known to overexpress GFP (see https://www.abcam.com/products/primary-antibodies/gfp-antibody-ab5450.html).
Additionally, a negative control has always been included in all analysis to ensure the specificity of the antibody.

Eukaryotic cell lines

Policy information about cell lines and Sex and Gender in Research

Cell line source(s)	N/A
Authentication	N/A
Mycoplasma contamination	N/A
Commonly misidentified lines (See ICLAC register)	N/A

Palaeontology and Archaeology

Specimen provenance	N/A
Specimen deposition	N/A
Dating methods	N/A

[ ] Tick this box to confirm that the raw and calibrated dates are available in the paper or in Supplementary Information.

Ethics oversight	N/A

Note that full information on the approval of the study protocol must also be provided in the manuscript.

Animals and other research organisms

Policy information about studies involving animals; ARRIVE guidelines recommended for reporting animal research, and Sex and Gender in Research

Laboratory animals	- RNAi line white CG2759 BDSC_35573 y[1] sc[*] v[1] sev[21]; P{y[+t7.7] v[+t1.8]=TRiP.GL00094}attP2<br>- RNAi line armitage CG11513 BDSC_34789 y[1] sc[*] v[1] sev[21]; P{y[+t7.7] v[+t1.8]=TRiP.HMS00098}attP2 from Bloomington<br>- RNAi line yb CG6272 VDRC_110056 P{KK105851}VIE-260B from VDRC<br>- RNAi line piwi CG6122 BDSC_37483 y[1] sc[*] v[1] sev[21]; P{y[+t7.7] v[+t1.8]=TRiP.GL00626}attP40 from Bloomington<br>- RNAi line vreteno CG4771 VDRC_34897 w1118; P{GD11424}v34897 from VDRC<br>- RNAi line zucchini CG12314 BDSC_35227 y[1] sc[*] v[1] sev[21]; P{y[+t7.7] v[+t1.8]=TRiP.GL00111}attP2<br>- RNAi line argonaute 3 CG40300 y[1] sc[*] v[1] sev[21]; P{y[+t7.7] v[+t1.8]=TRiP.GL00117}attP2<br>- white mutant, otherwise wild-type white BDSC_3605 w[1118] from Bloomington<br>- nanos-Gal4 driver BDSC_4937 w[1118];P{w[+mC]=GAL4::VP16nos.UTR}CG6325[MVD1] from Bloomington<br>- traffic-jam-Gal4 driver w; tj-Gal4/CyO<br>- RevI-H2i2 Produced in the lab Isogenic line derived from the RevI-H2 stock<br>All analyzed Drosophila flies were 3-6 days old.
Wild animals	The study did not involve wild animals
Reporting on sex	Studies were performed on females
Field-collected samples	This study involved no fiels-collected samples
Ethics oversight	No ethical approval was required for studies using Drosophila

Note that full information on the approval of the study protocol must also be provided in the manuscript.

Clinical data

Policy information about clinical studies

All manuscripts should comply with the ICMJEguidelines for publication of clinical research and a completedCONSORT checklist must be included with all submissions.

Clinical trial registration	N/A
Study protocol	N/A
Data collection	N/A
Outcomes	N/A

Dual use research of concern

Policy information about dual use research of concern

Hazards

Could the accidental, deliberate or reckless misuse of agents or technologies generated in the work, or the application of information presented in the manuscript, pose a threat to:

No	Yes	
☐	☐	Public health
☐	☐	National security
☐	☐	Crops and/or livestock
☐	☐	Ecosystems
☐	☐	Any other significant area

Experiments of concern

Does the work involve any of these experiments of concern:

No	Yes	
☐	☐	Demonstrate how to render a vaccine ineffective
☐	☐	Confer resistance to therapeutically useful antibiotics or antiviral agents
☐	☐	Enhance the virulence of a pathogen or render a nonpathogen virulent
☐	☐	Increase transmissibility of a pathogen
☐	☐	Alter the host range of a pathogen
☐	☐	Enable evasion of diagnostic/detection modalities
☐	☐	Enable the weaponization of a biological agent or toxin
☐	☐	Any other potentially harmful combination of experiments and agents

Plants

Seed stocks	N/A
Novel plant genotypes	N/A
Authentication	N/A

ChIP-seq

Data deposition

☐ Confirm that both raw and final processed data have been deposited in a public database such as GEO.

☐ Confirm that you have deposited or provided access to graph files (e.g. BED files) for the called peaks.

Data access links<br>*May remain private before publication.*	*For "Initial submission" or "Revised version" documents, provide reviewer access links. For your "Final submission" document, provide a link to the deposited data.*
Files in database submission	*Provide a list of all files available in the database submission.*
Genome browser session<br>(e.g. UCSC)	*Provide a link to an anonymized genome browser session for "Initial submission" and "Revised version" documents only, to enable peer review. Write "no longer applicable" for "Final submission" documents.*

Methodology

Replicates	*Describe the experimental replicates, specifying number, type and replicate agreement.*
Sequencing depth	*Describe the sequencing depth for each experiment, providing the total number of reads, uniquely mapped reads, length of reads and whether they were paired- or single-end.*
Antibodies	*Describe the antibodies used for the ChIP-seq experiments; as applicable, provide supplier name, catalog number, clone name, and lot number.*
Peak calling parameters	*Specify the command line program and parameters used for read mapping and peak calling, including the ChIP, control and index files used.*
Data quality	*Describe the methods used to ensure data quality in full detail, including how many peaks are at FDR 5% and above 5-fold enrichment.*
Software	*Describe the software used to collect and analyze the ChIP-seq data. For custom code that has been deposited into a community repository, provide accession details.*

Flow Cytometry

Plots

Confirm that:

☐ The axis labels state the marker and fluorochrome used (e.g. CD4-FITC).

☐ The axis scales are clearly visible. Include numbers along axes only for bottom left plot of group (a 'group' is an analysis of identical markers).

☐ All plots are contour plots with outliers or pseudocolor plots.

☐ A numerical value for number of cells or percentage (with statistics) is provided.

Methodology

Sample preparation	*Describe the sample preparation, detailing the biological source of the cells and any tissue processing steps used.*
Instrument	*Identify the instrument used for data collection, specifying make and model number.*
Software	*Describe the software used to collect and analyze the flow cytometry data. For custom code that has been deposited into a community repository, provide accession details.*
Cell population abundance	*Describe the abundance of the relevant cell populations within post-sort fractions, providing details on the purity of the samples and how it was determined.*
Gating strategy	*Describe the gating strategy used for all relevant experiments, specifying the preliminary FSC/SSC gates of the starting cell population, indicating where boundaries between "positive" and "negative" staining cell populations are defined.*

☐ Tick this box to confirm that a figure exemplifying the gating strategy is provided in the Supplementary Information.

Magnetic resonance imaging

Experimental design

Design type	*Indicate task or resting state; event-related or block design.*
Design specifications	*Specify the number of blocks, trials or experimental units per session and/or subject, and specify the length of each trial or block (if trials are blocked) and interval between trials.*
Behavioral performance measures	*State number and/or type of variables recorded (e.g. correct button press, response time) and what statistics were used to establish that the subjects were performing the task as expected (e.g. mean, range, and/or standard deviation across subjects).*

Acquisition

Imaging type(s)	*Specify: functional, structural, diffusion, perfusion.*
Field strength	*Specify in Tesla*
Sequence & imaging parameters	*Specify the pulse sequence type (gradient echo, spin echo, etc.), imaging type (EPI, spiral, etc.), field of view, matrix size, slice thickness, orientation and TE/TR/flip angle.*
Area of acquisition	*State whether a whole brain scan was used OR define the area of acquisition, describing how the region was determined.*

Diffusion MRI ☐ Used ☐ Not used

Preprocessing

Preprocessing software	*Provide detail on software version and revision number and on specific parameters (model/functions, brain extraction, segmentation, smoothing kernel size, etc.).*
Normalization	*If data were normalized/standardized, describe the approach(es): specify linear or non-linear and define image types used for transformation OR indicate that data were not normalized and explain rationale for lack of normalization.*
Normalization template	*Describe the template used for normalization/transformation, specifying subject space or group standardized space (e.g. original Talairach, MNI305, ICBM152) OR indicate that the data were not normalized.*
Noise and artifact removal	*Describe your procedure(s) for artifact and structured noise removal, specifying motion parameters, tissue signals and physiological signals (heart rate, respiration).*
Volume censoring	*Define your software and/or method and criteria for volume censoring, and state the extent of such censoring.*

Statistical modeling & inference

Model type and settings	*Specify type (mass univariate, multivariate, RSA, predictive, etc.) and describe essential details of the model at the first and second levels (e.g. fixed, random or mixed effects; drift or auto-correlation).*
Effect(s) tested	*Define precise effect in terms of the task or stimulus conditions instead of psychological concepts and indicate whether ANOVA or factorial designs were used.*

Specify type of analysis: ☐ Whole brain ☐ ROI-based ☐ Both

Statistic type for inference (See Eklund et al. 2016)	*Specify voxel-wise or cluster-wise and report all relevant parameters for cluster-wise methods.*

Correction | *Describe the type of correction and how it is obtained for multiple comparisons (e.g. FWE, FDR, permutation or Monte Carlo).*

Models & analysis

n/a	Involved in the study
☐	☐ Functional and/or effective connectivity
☐	☐ Graph analysis
☐	☐ Multivariate modeling or predictive analysis

Functional and/or effective connectivity | *Report the measures of dependence used and the model details (e.g. Pearson correlation, partial correlation, mutual information).*

Graph analysis | *Report the dependent variable and connectivity measure, specifying weighted graph or binarized graph, subject- or group-level, and the global and/or node summaries used (e.g. clustering coefficient, efficiency, etc.).*

Multivariate modeling and predictive analysis | *Specify independent variables, features extraction and dimension reduction, model, training and evaluation metrics.*
